# Supplementary material for: Empirical methods for controlling false positives and estimating confidence in ChIP-Seq peaks
Source: BMC Bioinformatics. 2008 Dec 5;9:523. doi: 10.1186/1471-2105-9-523 (PMC2628906; doi:10.1186/1471-2105-9-523)
Supplement: Additional File 1 — A variety of html documents from the USeq web site detailing the available applications, their best usage, output file type descriptions, command line menus, etc. [file 1471-2105-9-523-S1.zip › USeqUserGuides/JavaDoc.zip!/JavaDoc/util/gen/Num.html]

Num


|  |  |  |
| --- | --- | --- |
| **Overview** \| **Package** \| **Class** \| **Use** \| **Tree** \| **Deprecated** \| **Index** \| **Help** | |  |
| **PREV CLASS**   **NEXT CLASS** | **FRAMES**    **NO FRAMES**     **All Classes** |  |
| SUMMARY: NESTED \| FIELD \| CONSTR \| METHOD | DETAIL: FIELD \| CONSTR \| METHOD |  |


---


## util.gen Class Num

```
java.lang.Object
  util.gen.Num
```

---

``` public class Num extends java.lang.Object ```

Math and statistical static methods.

---

| **Field Summary** | |
| --- | --- |
| `static double` | `log10` |
| `static double` | `log2` |


**Constructor Summary**

`Num()`


| **Method Summary** | |
| --- | --- |
| `static double` | `antiLog(double loggedValue, double base)`             Converts to unlogged value. |
| `static float[]` | `antiLog(float[] loggedValues, int base)`             Converts an array float[] to unlogged values. |
| `static double[]` | `appendDouble(double[] d, double newValue)`             Returns a new array with the newValue appended on the end. |
| `static int[][]` | `arrayList2IntArrayArray(java.util.ArrayList al)`             Converts an ArrayList of int[]s to int[][]. |
| `static double[]` | `arrayListOfDoubleToArray(java.util.ArrayList dbl)`             ArrayList of Double to double[] |
| `static float[]` | `arrayListOfFloatToArray(java.util.ArrayList flt)`             ArrayList of Float to float[] |
| `static int[]` | `arrayListOfIntegerToInts(java.util.ArrayList integers)`             ArrayList of Integer to int[] |
| `static double[]` | `arrayListToDoubles(java.util.ArrayList doubles)`             ArrayList of Double to double[] |
| `static double` | `aveFoldDiffCombine(int[] t, int[] c)`             Calculates the average fold difference between two int[]s of the same length. |
| `static double` | `aveFoldDiffIndividual(int[] t, int[] c)`             Calculates the average fold difference between two int[]s of the same length. |
| `static double` | `averageFloatArray(float[] f)`             Averages a float[] |
| `static double[]` | `averageFloatArrays(float[][] ints)`             Average float[][] values to double[], averages repeats [values][numRepeats] |
| `static double[]` | `averageFloatArraysFlipped(float[][] ints)`             Average float[][] values to double[], averages repeats [numChips][OligoValues]. |
| `static float[]` | `averageFloatArraysFlippedToFloat(float[][] ints)`             Average float[][] values to double[], averages repeats [numChips][OligoValues]. |
| `static float[]` | `averageFloatArraysFlippedToFloats(float[][] ints)`             Average float[][] values to float[], averages repeats [numChips][OligoValues]. |
| `static double` | `averageIntArray(int[] f)`             Averages a int[] |
| `static double[]` | `averageIntIntArray(int[][] ints)`             Average int[][] values to double[], averages repeats [numRepeats][values] |
| `static double[]` | `averageIntIntArray2(int[][] ints)`             Average int[][] values to double[], averages values [numRepeats][values] |
| `static double[]` | `binomialPValues(double expect, java.io.File tempDirectory, int[][] treatObsControlObs, java.io.File fullPathToR)`             Calculates p-values using a binomial probablility from built in R function. |
| `static double` | `calcMaxMedianAbsoluteDifference(float[][] oligosValues)`             Calculates the maximum median absolute difference between arrays of float where int[oligo index number][oligo intensity measurements] |
| `static double` | `calcMeanMedianAbsoluteDifference(float[][] oligosValues)`             Calculates the mean of the median absolute differences between arrays of float where float[oligo index number][oligo intensity measurements] |
| `static int` | `calculateMiddleIntergenicCoordinates(int start, int end)`             Using interbase coordinates so length = end - start. |
| `static double[]` | `collapseDoubleArray(double[][] fs)`             Assumes equal lengths of the double[]s |
| `static float[]` | `collapseFloatArray(float[][] fs)`             Assumes equal lengths of the float[]s |
| `static int[]` | `collapseIntArray(int[][] ints)`             Any length OK of the int[]s |
| `static float[]` | `collapsePartOfAnArray(float[][] fs, int startIndex, int stopIndex)`             Assumes equal lengths of the float[]s |
| `static float[]` | `concatinate(float[][] f)`             Concatinates the float[]s, variable sizes OK. |
| `static float[]` | `concatinate(float[] one, float[] two)`             Joins two float[]s using System.arraycopy(). |
| `static double[]` | `convertToDouble(int[] d)`             Convert to double[] |
| `static float[]` | `convertToFloat(double[] d)`             Converts a double[] to float[] |
| `static float[]` | `convertToFloat(int[] d)`             Convert to float[] |
| `static int[]` | `convertToInt(double[] d)`             Convert to int[] |
| `static int[]` | `convertToInt(float[] d)`             Convert to int[] |
| `static double[]` | `convertToMedianZScores(double[] values)`             Converts an array of values to z scores using median as appose to mean. |
| `static void` | `convertToNeg10Log10(double[] d)`             Converts an array of double using -10 \* (Math.log(double)/log10). |
| `static double[]` | `convertToZScores(double[] values)`             Converts an array of values to z scores. |
| `static double[]` | `convertToZScores(double[] values, double mean, double stndDev)`             Converts an array of values to z scores given their mean and standard deviation. |
| `static double` | `correlationCoefficient(int[] x, int[] y)`             Calculates Pearson correlation coefficient, r, from two int[]s. |
| `static int` | `countNumberTrue(boolean[] b)` |
| `static int` | `countObjects(java.lang.Object[][] i)`             Returns the number of objects in the arrays. |
| `static int` | `countOutliers(float[] f, double threshold, boolean exceedsThreshold)`             Counts the number of values that are > or < threshold based on boolean. |
| `static int` | `countValues(int[][] i)`             Returns the number of elements/ values in the array. |
| `static double[]` | `difference(double[] t, double[] c)`             Returns the difference of each pair. |
| `static float[]` | `difference(float[][] t, float[][] c)`             Returns the difference of each pair of average, aveT - aveC. |
| `static double[]` | `difference(int[] t, int[] c)`             Returns the difference of each pair. |
| `static java.lang.String` | `doubleArrayToString(double[] d, int numberOfDecimalPlaces, java.lang.String delimiter)`             Converts an array of double to a String with a defined number of decimal places and a delimiter. |
| `static java.lang.String` | `doubleArrayToStringOnlyMax(double[] d, int maxNumDec, java.lang.String delimiter)`             Converts an array of double to a String with a maximal number of defined decimal places and a delimiter. |
| `static float[][]` | `expandFloatArray(float[] f, int numRows, int numColumns)`             Converts a float[] to float[][], must be certain about the number of rows and colmns! |
| `static double` | `factorial(int n)`             Calculates N! modified from http://www.unix.org.ua/orelly/java-ent/jnut/ch01\_03.htm Good for n < 21. |
| `static double` | `factorialApproximation(double n)`             Srivivasa Ramanuja ln(n!) factorial estimation, better than Stirling's approximation. |
| `static float[]` | `fetchMatrixValues(float[][] matrix, int[][] xy)`             Given a square table and a list of coordinates, will return the associated values. |
| `static int` | `findClosestEndIndex(int[] positions, int key)`             Finds the index of the key in the int[] but wont exceed the int[index] |
| `static int` | `findClosestIndexToValue(float[] sortedValues, float key)`             Returns the index of the closest values[index] to the key. |
| `static int` | `findClosestIndexToValue(int[] sortedValues, int key)`             Returns the index of the closest values[index] to the key. |
| `static int` | `findClosestStartIndex(int[] positions, int key)`             Finds the index of the key in the int[] but wont preceed the int[index] |
| `static float` | `findHighestFloat(float[] ints)`             Finds and returns the biggest float in an float[]. |
| `static int` | `findHighestInt(int[] ints)`             Finds and returns the biggest int in an int[]. |
| `static int` | `findMaxIntIndex(int[] ints)`             Returns the first index containing the maximum value. |
| `static double[]` | `findMinMaxDoubleValues(double[] f)`             Finds min and max values of a float array. |
| `static float[]` | `findMinMaxFloatArrays(float[][] f)`             Finds min and max values of a float array. |
| `static float[]` | `findMinMaxFloatValues(float[] f)`             Finds min and max values of an unsorted float array. |
| `static int[]` | `findMinMaxIntInt(int[][] f)`             Finds min and max values of a int array. |
| `static int[]` | `findMinMaxIntValues(int[] f)`             Finds min and max values of a int array. |
| `static int[]` | `findStartStopIndexes(int[] positions, int startValue, int stopValue, boolean notExact)`             Finds the start and stop indexes given a sorted int[] of values and two values, one after the other, close together, otherwise just use Arrays.binarySearch. |
| `static boolean` | `findZeros(float[] f)`             Scans a float array for zeros, if found returns true, other wise returns false. |
| `static java.lang.String` | `floatArrayToString(float[] f, java.lang.String delimiter)`             Converts to String using delimiter. |
| `static java.lang.String` | `formatNumber(double num, int numberOfDecimalPlaces)`             Converts a double ddd.dddddddd to a user determined number of decimal places right of the . |
| `static java.lang.String` | `formatNumberOneFraction(double num)`             Converts a double ddd.dddddddd to sss.s |
| `static java.lang.String` | `formatPercentOneFraction(double num)`             Converts 0.345543 to 34.6% |
| `static double[]` | `geoMeanFloatArraysFlipped(float[][] ints)`             Takes the geometric mean of float[][] values to double[], [numChips][OligoValues], returns the values in log base 2. |
| `static double` | `geometricMean(double[] x)`             Geometric mean. |
| `static double[]` | `geometricMean(float[][] ints)`             Takes a geometric average of float[][] values [numChips][OligoValues]. |
| `static float[]` | `geometricMeanRatio(float[][] t, float[][] c)`             Returns the log2 ratio of each pair, takes a geometric mean of replicas. |
| `static double[]` | `geometricMeanSkipZeros(float[][] ints)`             Takes a geometric average of float[][] values to double[], averages repeats [numChips][OligoValues]. |
| `static float` | `getAverageInts(int start, int end)`             Gets the average of the integers bracketed and including the start and end. |
| `static float[]` | `hashSetToFloat(java.util.HashSet<java.lang.Float> hash)`             Given a HashSet of Floats returns an array of float. |
| `static int[]` | `hashSetToInt(java.util.HashSet<java.lang.Integer> hash)`             Given a HashSet of Integers returns an array of int. |
| `static float[][]` | `identifyOutliers(float[][] f, int[][] controls, float threshold, boolean exceedsThreshold)`             Loads a zeroed matrix with values that are > or < threshold based on boolean. |
| `static java.lang.String` | `intArrayToString(int[] d, java.lang.String delimiter)`             Converts an array of int to a String seperated by the delimiter. |
| `static void` | `invertArray(float[] x)`             Inverts the float[]. |
| `static double[]` | `layeredLogRatios(float[][] oligoTreat, float[][] oligoCont)`             Given two float[replica][values] for treat and cont, for each replica makes all pairwise log2 ratios between treat and control. |
| `static float[][]` | `layeredLogRatiosSeperate(float[][] treatment, float[][] control)`             Given two float[replica][intensities] for treat and cont, for each oligo makes and returns all pairwise log2 ratios between treat and control replicas. |
| `static double[]` | `layeredRatios(float[][] oligoTreat, float[][] oligoCont)`             Given two float[replica][values] for treat and cont, for each replica makes all pairwise ratios between treat and control. |
| `static float[][]` | `layeredRatiosSeperate(float[][] treatment, float[][] control)`             Given two float[replica][intensities] for treat and cont, for each oligo makes and returns all pairwise ratios between treat and control replicas. |
| `static double[]` | `layeredRelativeDifferences(float[][] oligoTreat, float[][] oligoCont)`             Given two float[replica][values] for treat and cont, for each replica makes all pairwise relative differences between treat and control. |
| `static float[][]` | `layeredRelativeDifferencesSeperate(float[][] treatment, float[][] control)`             Given two float[replica][intensities] for treat and cont, for each oligo makes and returns all pairwise relative differences between treat and control replicas. |
| `static double` | `ln2Log10(double ln)`             Convert natural log to log 10. |
| `static double` | `ln2Minus10Log10(double ln)`             Convert natural log to log 10. |
| `static double[][]` | `loadDoubleMatrix(java.io.File f)`             Loads matrix of double[row number][double columns], white space delimited. |
| `static double[]` | `loadDoubles(java.io.File f)`             Loads a column of double from a file into an array. |
| `static java.util.LinkedHashMap` | `loadDoubles(java.io.File dataFile1, boolean convertLog10ToLog2)`             Loads columns of double into a HashMap, the first row is parsed as Strings and used as the key. |
| `static float[][]` | `loadFloatArray(int x, int y, float value)`             Makes a float[x][y] and fills it with the value. |
| `static float[]` | `loadFloats(java.io.File f)`             Loads a column of float from a file into an array. |
| `static int[]` | `loadInts(java.io.File f)`             Loads a column of ints from a file into an array. |
| `static float[][]` | `loadMatrixValues(float[][] matrix, int[][] xy)`             Given a square table and a list of coordinates, will return the associated values in a new zeroed square table. |
| `static double` | `log10(double number)`             Returns the LOG base 10 of the number. |
| `static double` | `log2(double number)`             Returns the LOG base 2 of the number. |
| `static void` | `log2(double[] f)`             Log2s every number in the array replacing the original values. |
| `static float` | `log2(float number)`             Returns the LOG base 2 of the number. |
| `static void` | `log2(float[] f)`             Log2s every number in the array replacing the original values. |
| `static void` | `log2(float[][] f)`             Log2s every number in the array replacing the original values. |
| `static float[]` | `log2Return(float[] f)`             Log2s every number in the array keeping original intact. |
| `static float[][]` | `log2Return(float[][] f)`             Log2s every number in the array returning the values, keep original intact. |
| `static double[]` | `log2ReturnDouble(float[] f)`             Log2s every number in the array keeping original intact. |
| `static double[]` | `logRatios(double[] t, double[] c)`             Calculates log base 2 ratios. |
| `static float[]` | `logRatios(float[][] t, float[][] c)`             Returns the log2 ratio of each pair, averaging replicas. |
| `static double[]` | `logRatios(float[] t, float[] c)`             Calculates log base 2 ratios. |
| `static void` | `maskOutliers(float[][] f, float outlierThreshold)`             Runs through float[][] setting any values that exceed the outlierThreshold to zero. |
| `static double` | `matScore(float[] t, float[] c)`             Takes unlogged MAT normalized t-values, sorts, logs and takes a variant of a number stabilized trimmed mean ratio. |
| `static double` | `mean(double[] t)`             Averages a double array. |
| `static float` | `mean(float[] t)`             Averages a float array. |
| `static float[][]` | `mean(float[][] one, float[][] two)`             Averages two float[][]s, assumes a square. |
| `static float[]` | `mean(float[] one, float[] two)`             Averages two float[]s . |
| `static double` | `mean(int[] t)`             Averages a int array. |
| `static java.lang.String` | `meanDoubles(java.util.ArrayList Doubles)`             Averages an ArrayList of Double objects. |
| `static double` | `meanIgnoreNaNInfinity(float[] f)`             Calculates a mean on the non NaN and Infinity floats, return zero if no non zero values were found. |
| `static int` | `meanIntegers(java.util.ArrayList Integers)`             Averages an ArrayList of Integer objects. |
| `static double` | `median(double[] sorted)`             Calculates Median of a sorted double[]. |
| `static double` | `median(float[] sorted)`             Calculates Median of a sorted float[]. |
| `static double` | `median(int[] sorted)`             Calculates Median of a sorted int[]. |
| `static double` | `median(short[] sorted)`             Calculates Median of a sorted short[]. |
| `static double` | `medianAbsoluteDifference(float[] x, float[] y)`             Calculates the median absolute difference. |
| `static java.lang.String` | `medianFloatArray(float[][] replicasIntensities)` |
| `static double` | `medianIgnoreZeros(float[] m)`             Calculates the median value of a sorted float[] ignoring leading zero values! |
| `static double[]` | `medianNormalize(double[] f, double targetMedian)`             Median normalize unsorted array to a given number. |
| `static float[][]` | `medianNormalize(float[][] f, double targetMedian)`             Median normalizes an unsorted float[][] array to a given target. |
| `static float[]` | `medianNormalize(float[] f, float targetMedian)`             Median normalize unsorted array to a given number. |
| `static double` | `millisecToDays(long ms)`             Converts milliseconds to days. |
| `static double` | `minus10log10(double pvalue)`             Returns the -10 \* LOG base 10 of the number. |
| `static int[]` | `modeOfHistogram(int[] ints)`             Finds mode of a int[] histogram array, assumes one peak, returns the index position, value/frequency. |
| `static double[]` | `pairedLogRatios(float[][] treatments, float[][] controls)`             Given two float[replica][values] for treat and cont, for each replica makes matched pairwise log ratios between treat and control. |
| `static double[]` | `pairedRatios(float[][] treatments, float[][] controls)`             Given two float[replica][values] for treat and cont, for each replica makes matched pairwise ratios between treat and control. |
| `static double[]` | `pairedRelativeDifferences(float[][] treatments, float[][] controls)`             Given two float[replica][values] for treat and cont, for each replica makes matched pairwise relative differences between treat and control. |
| `static float[]` | `pairwiseMeans(float[] d)`             Returns all pairwise averages. |
| `static double[]` | `pairwiseMeansDouble(float[] d)`             Returns all pairwise averages. |
| `static double[]` | `parseDoubles(java.lang.String[] doubles)`             Parses doubles from a String[] of doubles. |
| `static int` | `parseInt(java.lang.String stringInt, int failNumber)`             Attemps to parse an int, returns failNumber if it fails. |
| `static int[]` | `parseInts(java.lang.String[] ints)`             Parses ints from a String[] of ints. |
| `static double[]` | `partialPairwiseMeans(float[] a, float[] b)`             Returns all pairwise averages between a and b but not self pairwise. |
| `static float` | `percentile(float[] sortedFloats, double percentile)`             Returns the value of a given percentile from a SORTED array. |
| `static double` | `poissonProbabilityApproximation(double mean, int observed)`             Calculates a natural log approximation of the Poisson probablility. |
| `static double` | `poissonProbablility(double rate, int observed)`             Calculates the Poisson probablility for observed < 21. |
| `static double[]` | `poissonPValues(int maxHit, double rate, java.io.File fullPathToR, java.io.File tempDir, double bonCorr)`             Calculates p-values using a Poisson probablility from built in R function. |
| `static float[][]` | `pooledSubArray(float[][] treatment, float[][] control, int startIndex, int stopIndex)`             Builds a pooled from part of a float[replica][intensities] for treat and control. |
| `static double[]` | `prependDouble(double[] d, double newValue)`             Returns a new array with the newValue appended on the beginning. |
| `static double` | `pseudoMedian(double[] d)`             Returns a Hodges-Lehmann estimator, the median of all pairwise means. |
| `static double` | `pseudoMedian(float[] d)`             Returns a Hodges-Lehmann estimator, the median of all pairwise means. |
| `static double[]` | `pseudoMedian(float[][] repInt)`             Calculates the pseudoMedian on each replica of intensities. |
| `static double[]` | `quartiles(float[] sortedFloatArray)`             Calculates the Q1,Q2,and Q3 (25th, 50th, and 75th percentile). |
| `static void` | `randomize(float[][] f)`             Randomizes the intensities, not between replicas. |
| `static void` | `randomize(float[] f, long seed)`             Randomizes the array by permutating indexes and swapping. |
| `static void` | `randomizeFirstArray(float[][] f, long seed)`             Randomizes the first array by permutating indexes and swapping. |
| `static float[]` | `randomSample(float[] x, int numToSample)`             Returns an array of float[numToSample] populated with randomly picked values from x. |
| `static double[]` | `ratio(double[] t, double[] c)`             Returns the ratio of each pair. |
| `static float[]` | `ratio(float[][] t, float[][] c)`             Returns the ratio of each pair, averages replicas. |
| `static double[]` | `ratio(float[] t, float[] c)`             Returns the ratio of each pair. |
| `static double[]` | `ratio(int[] t, double[] c)`             Returns the ratio of each pair. |
| `static double[]` | `ratio(int[] t, int[] c)`             Returns the ratio of each pair. |
| `static int[][]` | `readBinaryInt2Array(java.io.File file)`             Reads a binary int[][2] array file. |
| `static double` | `relativeDifference(double t, double c)`             Calculates a relative difference. |
| `static float[]` | `relativeDifferences(float[][] t, float[][] c)`             Returns the relative difference of each pair, averages replicas. |
| `static double[]` | `relativeDifferences(float[] t, float[] c)`             Calculates relative differences. |
| `static double` | `relativeDifferenceToLog2Ratio(double relDiff)`             Converter |
| `static float[]` | `relativeDifferenceToLog2Ratio(float[] relDiff)`             Converter |
| `static double[]` | `removeNaNInfinite(double[] d)`             Removes any NaN or Infinite |
| `static float[]` | `removeZeroValues(float[] f)`             Remove zero values from float array. |
| `static float[][]` | `removeZeroValues(float[][] f)`             Provide a float[2][0], will remove zeros from both and there matched pair. |
| `static float[][][]` | `removeZeroValues(float[][] t, float[][] c)`             Removes zero values and their assoicated values from all arrays. |
| `static float[][]` | `rotateClockwise(float[][] matrix)`             Rotates a square matrix 90 degrees Clock Wise? |
| `static float[][]` | `rotateCounterClockwise(float[][] matrix)`             Rotates a square matrix 90 degrees CounterClockWise? |
| `static double[]` | `round(double[] d, int numDecimals)`             Rounds a double[] based on number of desired decimals, ie 1 = x.x, 2 = x.xx limited to 6 |
| `static double` | `round(double d, int numDecimals)`             Rounds a double based on number of desired decimals, ie 1 = x.x, 2 = x.xx limited to 6 |
| `static float[]` | `sam(float[] t, float[] c, double fudge)`             Calculates the SAM d statistic, similar to a t-statistic with a bit of fudge added into the denominator to control for zero variance conditions. |
| `static void` | `saveMatrixValues(float[][] matrix, int[][] xy, float threshold, java.io.File f)`             Given a square table and a list of coordinates, save the associated values. |
| `static void` | `scale1To1000(float[] f)`             Scales the array to 1:1000 |
| `static double[]` | `smoothScores(double[] scores, int[] positions, int windowSize)`             Uses a sliding window to smooth the scores applying a trimmed mean (drop 1 from ends) to the scores found within the window. |
| `static float[]` | `squareRoots(int num)`             Returns the integer square root from zero to num. |
| `static double` | `standardDeviation(double[] x)`             Calculates standard deviation of an double[] |
| `static double` | `standardDeviation(double[] x, double mean)`             Calculates standard deviation of a double[] and mean. |
| `static double` | `standardDeviation(float[] x)`             Calculates standard deviation of an float[] |
| `static double` | `standardDeviation(float[] x, double mean)`             Calculates standard deviation of an float[] given a mean. |
| `static double` | `standardDeviation(int[] x)`             Calculates standard deviation of an int[] |
| `static double` | `standardDeviation(int[] x, double mean)`             Calculates standard deviation of an int[] and mean. |
| `static double` | `standardDeviationOfDifference(int[] t, int[] c)`             Calculates the standard deviation of the difference between two int[]s of the same length. |
| `static double` | `standardError(double[] x, double mean)`             Calculates standard error of a double[] given it's mean. |
| `static void` | `statFloatArray(float[] f, boolean printHistogram)`             Calculates Min, Max, Mean, Median, Mode, and Histogram/10 for a Float[] |
| `static float[]` | `stringArrayToFloat(java.lang.String s, java.lang.String delimiter)`             Given a String of floats delimited by something, will parse or return null. |
| `static int[]` | `stringArrayToInts(java.lang.String s, java.lang.String delimiter)`             Given a String of ints delimited by something, will parse or return null. |
| `static float[][]` | `subArray(float[][] intensities, int startIndex, int stopIndex)`             Builds a sub array from part of a float[replica][intensities]. |
| `static float` | `sumArray(float[] array)`             Sums a float array |
| `static int` | `sumIntArray(int[] ints)`             Sums an int array |
| `static double` | `trimmedMean(double[] sortedFloat, double fraction)`             Trims the fraction off the top and bottom, returns the mean of the remainder, rounds trimming # up. |
| `static double` | `trimmedMean(double[] sortedDouble, int trimNumber)`             Makes a trimmed mean, note the trim number is not a % but the number of values to drop from the beginning and end of the ordered set. |
| `static double` | `trimmedMean(float[] sortedFloat, double fraction)`             Trims the fraction off the top and bottom, returns the mean of the remainder, rounds trimming # up. |
| `static double` | `trimmedMean(float[] sortedFloat, int trimNumber)`             Makes a trimmed mean, note the trim number is not a % but the number of values to drop from the beginning and end of the ordered set. |
| `static float[][]` | `trimOutliers(float[][] f, float cutoff, float replacement)`             For each f[replica][]Sets values exceeding the cutoff to the default. |
| `static float[]` | `trimOutliers(float[] f, float cutoff, float replacement)`             Sets values exceeding the cutoff to the default. |
| `static double` | `tukeyBiWeight(double[] x)`             Calculates a one-step tukey biweight estimator using 5 as the tuning constant and adding 0.0001 to the denominator to avoid dividing by zero, Affy's params. |
| `static double[]` | `windowAverageScores(double[] scores, int windowSize)`             Slides a window along an array, one index at a time, averaging the contents. |
| `static float[]` | `windowAverageScores(float[] scores, int windowSize)`             Slides a window along an array, one index at a time, averaging the contents. |
| `static float[]` | `windowAverageScoresIgnoreScore(float[][] scores, float scoreToIgnore, int windowSize)`             Slides a window along an array, one index at a time, averaging the contents, ignores zero values. |
| `static float[]` | `windowAverageScoresIgnoreScore(float[] scores, float scoreToIgnore, int windowSize)`             Slides a window along an array, one index at a time, averaging the contents. |
| `static double[]` | `windowAverageScoresIgnoreZeros(double[] scores, int windowSize)`             Slides a window along an array, one index at a time, averaging the contents, ignores zero values. |
| `static boolean` | `writeBinaryInt2Array(int[][] c, java.io.File file)`             Writes a binary int[][2] array. |
| `static boolean` | `writeToFile(double[] doubles, java.io.File doubleFile)`             Writes a float array to file, one per line. |
| `static boolean` | `writeToFile(float[] floats, java.io.File floatFile)`             Writes a float array to file, one per line. |
| `static float[][]` | `zeroedFloatArray(int x, int y)`             Makes a float[x][y] and fills it with zeros. |

**Methods inherited from class java.lang.Object**

`equals, getClass, hashCode, notify, notifyAll, toString, wait, wait, wait`

**Field Detail**

### log10

```
public static final double log10
```

---


### log2

```
public static final double log2
```


**Constructor Detail**

### Num

```
public Num()
```


**Method Detail**

### calculateMiddleIntergenicCoordinates

```
public static int calculateMiddleIntergenicCoordinates(int start,
                                                       int end)
```

:   Using interbase coordinates so length = end - start.

---


### countNumberTrue

```
public static int countNumberTrue(boolean[] b)
```

:   **Returns:**: number of trues in boolean[]

---


### scale1To1000

```
public static void scale1To1000(float[] f)
```

:   Scales the array to 1:1000

---


### poissonProbablility

```
public static double poissonProbablility(double rate,
                                         int observed)
```

:   Calculates the Poisson probablility for observed < 21.
    For observed > 20 call poissonProbabilityApproximation()

    :   **Parameters:**: `rate` - - lambda, the average number of occurances: `observed` - - the actual number of occurances observed **Returns:**: poissonProbability of observing the number of occurances by chance alone

---


### poissonProbabilityApproximation

```
public static double poissonProbabilityApproximation(double mean,
                                                     int observed)
```

:   Calculates a natural log approximation of the Poisson probablility.

    :   **Parameters:**: `mean` - - lambda, the average number of occurances: `observed` - - the actual number of occurances observed **Returns:**: ln(poissonProbability) - the natural log of the poisson probability.

---


### ln2Log10

```
public static double ln2Log10(double ln)
```

:   Convert natural log to log 10.

---


### ln2Minus10Log10

```
public static double ln2Minus10Log10(double ln)
```

:   Convert natural log to log 10.

---


### relativeDifferenceToLog2Ratio

```
public static float[] relativeDifferenceToLog2Ratio(float[] relDiff)
```

:   Converter

---


### relativeDifferenceToLog2Ratio

```
public static double relativeDifferenceToLog2Ratio(double relDiff)
```

:   Converter

---


### millisecToDays

```
public static double millisecToDays(long ms)
```

:   Converts milliseconds to days.

---


### writeBinaryInt2Array

```
public static boolean writeBinaryInt2Array(int[][] c,
                                           java.io.File file)
```

:   Writes a binary int[][2] array.

    :   **Returns:**: true if sucessful, false if something bad happened.

---


### readBinaryInt2Array

```
public static int[][] readBinaryInt2Array(java.io.File file)
```

:   Reads a binary int[][2] array file.

    :   **Returns:**: null if something bad happened.

---


### invertArray

```
public static void invertArray(float[] x)
```

:   Inverts the float[].

---


### convertToFloat

```
public static float[] convertToFloat(double[] d)
```

:   Converts a double[] to float[]

---


### factorial

```
public static double factorial(int n)
```

:   Calculates N! modified from http://www.unix.org.ua/orelly/java-ent/jnut/ch01\_03.htm
    Good for n < 21.

---


### factorialApproximation

```
public static double factorialApproximation(double n)
```

:   Srivivasa Ramanuja ln(n!) factorial estimation, better than Stirling's approximation.
    Good for larger values of n.

    :   **Returns:**: ln(n!)

---


### arrayList2IntArrayArray

```
public static int[][] arrayList2IntArrayArray(java.util.ArrayList al)
```

:   Converts an ArrayList of int[]s to int[][].

---


### poissonPValues

```
public static double[] poissonPValues(int maxHit,
                                      double rate,
                                      java.io.File fullPathToR,
                                      java.io.File tempDir,
                                      double bonCorr)
```

:   Calculates p-values using a Poisson probablility from built in R function.
    Set bonCorr to 1 if you don't want to Bonferroni correct the pvalues.
    You should check that the pvalues don't exceed 1 if you do correct.

    :   **Returns:**: pvalues where the index is the observed number of hits.

---


### binomialPValues

```
public static double[] binomialPValues(double expect,
                                       java.io.File tempDirectory,
                                       int[][] treatObsControlObs,
                                       java.io.File fullPathToR)
```

:   Calculates p-values using a binomial probablility from built in R function.

---


### convertToNeg10Log10

```
public static void convertToNeg10Log10(double[] d)
```

:   Converts an array of double using -10 \* (Math.log(double)/log10).
    Any doubles <=0 are assigned the minimal double from the array >0 then transformed.

---


### hashSetToFloat

```
public static float[] hashSetToFloat(java.util.HashSet<java.lang.Float> hash)
```

:   Given a HashSet of Floats returns an array of float.

---


### hashSetToInt

```
public static int[] hashSetToInt(java.util.HashSet<java.lang.Integer> hash)
```

:   Given a HashSet of Integers returns an array of int.

---


### antiLog

```
public static double antiLog(double loggedValue,
                             double base)
```

:   Converts to unlogged value.

---


### antiLog

```
public static float[] antiLog(float[] loggedValues,
                              int base)
```

:   Converts an array float[] to unlogged values.

---


### findZeros

```
public static boolean findZeros(float[] f)
```

:   Scans a float array for zeros, if found returns true, other wise returns false.

---


### rotateCounterClockwise

```
public static float[][] rotateCounterClockwise(float[][] matrix)
```

:   Rotates a square matrix 90 degrees CounterClockWise?

---


### rotateClockwise

```
public static float[][] rotateClockwise(float[][] matrix)
```

:   Rotates a square matrix 90 degrees Clock Wise?

---


### findMaxIntIndex

```
public static int findMaxIntIndex(int[] ints)
```

:   Returns the first index containing the maximum value.

---


### log2

```
public static void log2(float[][] f)
```

:   Log2s every number in the array replacing the original values.

---


### log2Return

```
public static float[][] log2Return(float[][] f)
```

:   Log2s every number in the array returning the values, keep original intact.

---


### log2

```
public static void log2(float[] f)
```

:   Log2s every number in the array replacing the original values.

---


### log2

```
public static void log2(double[] f)
```

:   Log2s every number in the array replacing the original values.

---


### log2Return

```
public static float[] log2Return(float[] f)
```

:   Log2s every number in the array keeping original intact.

---


### log2ReturnDouble

```
public static double[] log2ReturnDouble(float[] f)
```

:   Log2s every number in the array keeping original intact.

---


### loadDoubles

```
public static java.util.LinkedHashMap loadDoubles(java.io.File dataFile1,
                                                  boolean convertLog10ToLog2)
```

:   Loads columns of double into a HashMap, the first row is parsed as Strings and used as the key.
    The value is a double[].
    Can convert log10 values in the files to log2 (for Agilent data).
    Skips blank lines.

---


### parseDoubles

```
public static double[] parseDoubles(java.lang.String[] doubles)
```

:   Parses doubles from a String[] of doubles. Returns null if a parsing error is encountered.

---


### parseInts

```
public static int[] parseInts(java.lang.String[] ints)
```

:   Parses ints from a String[] of ints. Returns null if a parsing error is encountered.

---


### convertToZScores

```
public static double[] convertToZScores(double[] values,
                                        double mean,
                                        double stndDev)
```

:   Converts an array of values to z scores given their mean and standard deviation.

---


### convertToZScores

```
public static double[] convertToZScores(double[] values)
```

:   Converts an array of values to z scores.

---


### convertToMedianZScores

```
public static double[] convertToMedianZScores(double[] values)
```

:   Converts an array of values to z scores using median as appose to mean.

---


### appendDouble

```
public static double[] appendDouble(double[] d,
                                    double newValue)
```

:   Returns a new array with the newValue appended on the end.

---


### prependDouble

```
public static double[] prependDouble(double[] d,
                                     double newValue)
```

:   Returns a new array with the newValue appended on the beginning.

---


### tukeyBiWeight

```
public static double tukeyBiWeight(double[] x)
```

:   Calculates a one-step tukey biweight estimator using
    5 as the tuning constant and adding 0.0001 to the denominator
    to avoid dividing by zero, Affy's params.
    If all zeros are submitted then returns zero.

---


### geometricMean

```
public static double geometricMean(double[] x)
```

:   Geometric mean. Cannot have any zeros or negative numbers in the double[].

---


### percentile

```
public static float percentile(float[] sortedFloats,
                               double percentile)
```

:   Returns the value of a given percentile from a SORTED array.
    Percentile is from 0-1, ie 0.95 and is according to Lentner, 1982.

---


### maskOutliers

```
public static void maskOutliers(float[][] f,
                                float outlierThreshold)
```

:   Runs through float[][] setting any values that exceed the outlierThreshold
    to zero. Assumes float[i]s are equal length.

---


### countValues

```
public static int countValues(int[][] i)
```

:   Returns the number of elements/ values in the array.

---


### countObjects

```
public static int countObjects(java.lang.Object[][] i)
```

:   Returns the number of objects in the arrays.

---


### countOutliers

```
public static int countOutliers(float[] f,
                                double threshold,
                                boolean exceedsThreshold)
```

:   Counts the number of values that are > or < threshold based on boolean.
    Returns - number of outliers > or < threshold
    Param - exceedsThreshold, boolean flag when true counts the number that
    exceed the threshold, when false the number less than the threshold.

---


### identifyOutliers

```
public static float[][] identifyOutliers(float[][] f,
                                         int[][] controls,
                                         float threshold,
                                         boolean exceedsThreshold)
```

:   Loads a zeroed matrix with values that are > or < threshold based on boolean.
    Returns a loaded zeroed matrix.
    Param exceedsThreshold, boolean flag when true returns the values that
    exceed the threshold, less than the threshold.

---


### fetchMatrixValues

```
public static float[] fetchMatrixValues(float[][] matrix,
                                        int[][] xy)
```

:   Given a square table and a list of coordinates, will return the associated values.

---


### saveMatrixValues

```
public static void saveMatrixValues(float[][] matrix,
                                    int[][] xy,
                                    float threshold,
                                    java.io.File f)
```

:   Given a square table and a list of coordinates, save the associated values.

---


### loadMatrixValues

```
public static float[][] loadMatrixValues(float[][] matrix,
                                         int[][] xy)
```

:   Given a square table and a list of coordinates, will return the associated values
    in a new zeroed square table.

---


### quartiles

```
public static double[] quartiles(float[] sortedFloatArray)
```

:   Calculates the Q1,Q2,and Q3 (25th, 50th, and 75th percentile).
    Ignores the middle value in calculating Q1 and Q3 if the array length is odd.
    Don't forget to sort the array!

---


### zeroedFloatArray

```
public static float[][] zeroedFloatArray(int x,
                                         int y)
```

:   Makes a float[x][y] and fills it with zeros.

---


### loadFloatArray

```
public static float[][] loadFloatArray(int x,
                                       int y,
                                       float value)
```

:   Makes a float[x][y] and fills it with the value.

---


### randomSample

```
public static float[] randomSample(float[] x,
                                   int numToSample)
```

:   Returns an array of float[numToSample] populated with randomly picked values from x.

---


### randomize

```
public static void randomize(float[] f,
                             long seed)
```

:   Randomizes the array by permutating indexes and swapping. Fast.
    Can set seedMultiplier or vary it to be sure and get diff

---


### randomizeFirstArray

```
public static void randomizeFirstArray(float[][] f,
                                       long seed)
```

:   Randomizes the first array by permutating indexes and swapping. Fast.
    Shuffles f[shuffle][].

---


### randomize

```
public static void randomize(float[][] f)
```

:   Randomizes the intensities, not between replicas.

    :   **Parameters:**: `float[replicas][intensities]` -

---


### layeredRatiosSeperate

```
public static float[][] layeredRatiosSeperate(float[][] treatment,
                                              float[][] control)
```

:   Given two float[replica][intensities] for treat and cont, for each oligo makes and returns all pairwise ratios
    between treat and control replicas.

    :   **Parameters:**: `float[replica][intensities]` - for treatment and control **Returns:**: float[oligo index][all t/c ratios]

---


### layeredRelativeDifferencesSeperate

```
public static float[][] layeredRelativeDifferencesSeperate(float[][] treatment,
                                                           float[][] control)
```

:   Given two float[replica][intensities] for treat and cont, for each oligo makes and returns all pairwise relative differences
    between treat and control replicas.

    :   **Parameters:**: `float[replica][intensities]` - for treatment and control **Returns:**: float[oligo index][all relative differences]

---


### layeredRelativeDifferences

```
public static double[] layeredRelativeDifferences(float[][] oligoTreat,
                                                  float[][] oligoCont)
```

:   Given two float[replica][values] for treat and cont, for each replica makes all pairwise relative differences
    between treat and control. Returns as a big pool.

---


### layeredLogRatiosSeperate

```
public static float[][] layeredLogRatiosSeperate(float[][] treatment,
                                                 float[][] control)
```

:   Given two float[replica][intensities] for treat and cont, for each oligo makes and returns all pairwise log2 ratios
    between treat and control replicas.

    :   **Parameters:**: `float[replica][intensities]` - for treatment and control **Returns:**: float[oligo index][all log2(t/c) ratios]

---


### layeredRatios

```
public static double[] layeredRatios(float[][] oligoTreat,
                                     float[][] oligoCont)
```

:   Given two float[replica][values] for treat and cont, for each replica makes all pairwise ratios
    between treat and control.

---


### pairedRatios

```
public static double[] pairedRatios(float[][] treatments,
                                    float[][] controls)
```

:   Given two float[replica][values] for treat and cont, for each replica makes matched pairwise ratios
    between treat and control. Must have equal number of replicas for both T and C.

---


### pairedRelativeDifferences

```
public static double[] pairedRelativeDifferences(float[][] treatments,
                                                 float[][] controls)
```

:   Given two float[replica][values] for treat and cont, for each replica makes matched pairwise relative differences
    between treat and control. Must have equal number of replicas for both T and C.

---


### layeredLogRatios

```
public static double[] layeredLogRatios(float[][] oligoTreat,
                                        float[][] oligoCont)
```

:   Given two float[replica][values] for treat and cont, for each replica makes all pairwise
    log2 ratios between treat and control.
    Zero ratios are assigned a log2 of 0.0000000001

---


### pairedLogRatios

```
public static double[] pairedLogRatios(float[][] treatments,
                                       float[][] controls)
```

:   Given two float[replica][values] for treat and cont, for each replica makes matched pairwise log ratios
    between treat and control. Must have equal number of replicas for both T and C.

---


### matScore

```
public static double matScore(float[] t,
                              float[] c)
```

:   Takes unlogged MAT normalized t-values, sorts, logs and takes a variant of a number stabilized trimmed mean ratio.
    Returns 1 if trimmed mean will zero the number of values.

---


### pseudoMedian

```
public static double pseudoMedian(double[] d)
```

:   Returns a Hodges-Lehmann estimator, the median of all pairwise means.

---


### squareRoots

```
public static float[] squareRoots(int num)
```

:   Returns the integer square root from zero to num.

---


### pairwiseMeans

```
public static float[] pairwiseMeans(float[] d)
```

:   Returns all pairwise averages.

---


### pairwiseMeansDouble

```
public static double[] pairwiseMeansDouble(float[] d)
```

:   Returns all pairwise averages.

---


### partialPairwiseMeans

```
public static double[] partialPairwiseMeans(float[] a,
                                            float[] b)
```

:   Returns all pairwise averages between a and b but not self pairwise.

---


### concatinate

```
public static float[] concatinate(float[][] f)
```

:   Concatinates the float[]s, variable sizes OK.

---


### concatinate

```
public static float[] concatinate(float[] one,
                                  float[] two)
```

:   Joins two float[]s using System.arraycopy().

---


### subArray

```
public static float[][] subArray(float[][] intensities,
                                 int startIndex,
                                 int stopIndex)
```

:   Builds a sub array from part of a float[replica][intensities].

    :   **Parameters:**: `float[replicas][intensities]` -

---


### pseudoMedian

```
public static double[] pseudoMedian(float[][] repInt)
```

:   Calculates the pseudoMedian on each replica of intensities. If only one intensity,
    sets that as the pseudo median.

    :   **Parameters:**: `repInt` - float[replica index][actual intensity values]

---


### pseudoMedian

```
public static double pseudoMedian(float[] d)
```

:   Returns a Hodges-Lehmann estimator, the median of all pairwise means.
    If only one pairwise mean, return the mean instead of the median on the means.

---


### removeZeroValues

```
public static float[][][] removeZeroValues(float[][] t,
                                           float[][] c)
```

:   Removes zero values and their assoicated values from all arrays.
    Pass two float[numChips][oligoValues] representing treatment and control chips.
    The num oligoValues should be the same, the numChips can vary between t and c.
    Returns float[2][][] representing t[][] and c[][].

---


### trimOutliers

```
public static float[] trimOutliers(float[] f,
                                   float cutoff,
                                   float replacement)
```

:   Sets values exceeding the cutoff to the default.

---


### trimOutliers

```
public static float[][] trimOutliers(float[][] f,
                                     float cutoff,
                                     float replacement)
```

:   For each f[replica][]Sets values exceeding the cutoff to the default.

---


### medianNormalize

```
public static float[] medianNormalize(float[] f,
                                      float targetMedian)
```

:   Median normalize unsorted array to a given number.

---


### medianNormalize

```
public static double[] medianNormalize(double[] f,
                                       double targetMedian)
```

:   Median normalize unsorted array to a given number.

---


### medianNormalize

```
public static float[][] medianNormalize(float[][] f,
                                        double targetMedian)
```

:   Median normalizes an unsorted float[][] array to a given target.

---


### medianIgnoreZeros

```
public static double medianIgnoreZeros(float[] m)
```

:   Calculates the median value of a sorted float[] ignoring leading zero values!

---


### convertToInt

```
public static int[] convertToInt(double[] d)
```

:   Convert to int[]

---


### convertToDouble

```
public static double[] convertToDouble(int[] d)
```

:   Convert to double[]

---


### convertToInt

```
public static int[] convertToInt(float[] d)
```

:   Convert to int[]

---


### convertToFloat

```
public static float[] convertToFloat(int[] d)
```

:   Convert to float[]

---


### relativeDifferences

```
public static double[] relativeDifferences(float[] t,
                                           float[] c)
```

:   Calculates relative differences. Uses 2 \* ( (t-c)/(t+c) )

---


### relativeDifference

```
public static double relativeDifference(double t,
                                        double c)
```

:   Calculates a relative difference. Uses 2 \* ( (t-c)/(t+c) )

---


### logRatios

```
public static double[] logRatios(double[] t,
                                 double[] c)
```

:   Calculates log base 2 ratios.

---


### logRatios

```
public static double[] logRatios(float[] t,
                                 float[] c)
```

:   Calculates log base 2 ratios.

---


### round

```
public static double[] round(double[] d,
                             int numDecimals)
```

:   Rounds a double[] based on number of desired decimals, ie 1 = x.x, 2 = x.xx
    limited to 6

---


### round

```
public static double round(double d,
                           int numDecimals)
```

:   Rounds a double based on number of desired decimals, ie 1 = x.x, 2 = x.xx
    limited to 6

---


### removeNaNInfinite

```
public static double[] removeNaNInfinite(double[] d)
```

:   Removes any NaN or Infinite

---


### log10

```
public static double log10(double number)
```

:   Returns the LOG base 10 of the number.

---


### log2

```
public static double log2(double number)
```

:   Returns the LOG base 2 of the number.

---


### log2

```
public static float log2(float number)
```

:   Returns the LOG base 2 of the number.

---


### minus10log10

```
public static double minus10log10(double pvalue)
```

:   Returns the -10 \* LOG base 10 of the number.

---


### loadDoubleMatrix

```
public static double[][] loadDoubleMatrix(java.io.File f)
```

:   Loads matrix of double[row number][double columns], white space delimited.
    # comment lines and blank lines ignored.

---


### loadInts

```
public static int[] loadInts(java.io.File f)
```

:   Loads a column of ints from a file into an array.
    Returns null if nothing found.
    Skips blank lines.

---


### loadDoubles

```
public static double[] loadDoubles(java.io.File f)
```

:   Loads a column of double from a file into an array.
    Returns null if nothing found.
    Skips blank lines.

---


### loadFloats

```
public static float[] loadFloats(java.io.File f)
```

:   Loads a column of float from a file into an array.
    Returns null if nothing found.
    Skips blank lines.

---


### writeToFile

```
public static boolean writeToFile(float[] floats,
                                  java.io.File floatFile)
```

:   Writes a float array to file, one per line.

---


### writeToFile

```
public static boolean writeToFile(double[] doubles,
                                  java.io.File doubleFile)
```

:   Writes a float array to file, one per line.

---


### arrayListOfIntegerToInts

```
public static int[] arrayListOfIntegerToInts(java.util.ArrayList integers)
```

:   ArrayList of Integer to int[]

---


### arrayListToDoubles

```
public static double[] arrayListToDoubles(java.util.ArrayList doubles)
```

:   ArrayList of Double to double[]

---


### parseInt

```
public static int parseInt(java.lang.String stringInt,
                           int failNumber)
```

:   Attemps to parse an int, returns failNumber if it fails.

---


### statFloatArray

```
public static void statFloatArray(float[] f,
                                  boolean printHistogram)
```

:   Calculates Min, Max, Mean, Median, Mode, and Histogram/10 for a Float[]

---


### removeZeroValues

```
public static float[] removeZeroValues(float[] f)
```

:   Remove zero values from float array.

---


### getAverageInts

```
public static float getAverageInts(int start,
                                   int end)
```

:   Gets the average of the integers bracketed and including the start and end.
    (ie 3,6 returns the average of 3+4+5+6/4= 4.5)

---


### collapseIntArray

```
public static int[] collapseIntArray(int[][] ints)
```

:   Any length OK of the int[]s

---


### medianFloatArray

```
public static java.lang.String medianFloatArray(float[][] replicasIntensities)
```

:   **Parameters:**: `replicasIntensities` - - float[replicaNumber][associatedIntensities] **Returns:**: comma delimited list of medians, one for each replica.

---


### collapseFloatArray

```
public static float[] collapseFloatArray(float[][] fs)
```

:   Assumes equal lengths of the float[]s

---


### collapsePartOfAnArray

```
public static float[] collapsePartOfAnArray(float[][] fs,
                                            int startIndex,
                                            int stopIndex)
```

:   Assumes equal lengths of the float[]s

---


### collapseDoubleArray

```
public static double[] collapseDoubleArray(double[][] fs)
```

:   Assumes equal lengths of the double[]s

---


### expandFloatArray

```
public static float[][] expandFloatArray(float[] f,
                                         int numRows,
                                         int numColumns)
```

:   Converts a float[] to float[][], must be certain about the number of rows and colmns!

---


### averageIntArray

```
public static double averageIntArray(int[] f)
```

:   Averages a int[]

---


### averageFloatArray

```
public static double averageFloatArray(float[] f)
```

:   Averages a float[]

---


### findMinMaxIntValues

```
public static int[] findMinMaxIntValues(int[] f)
```

:   Finds min and max values of a int array.

---


### findMinMaxFloatValues

```
public static float[] findMinMaxFloatValues(float[] f)
```

:   Finds min and max values of an unsorted float array.

---


### findMinMaxDoubleValues

```
public static double[] findMinMaxDoubleValues(double[] f)
```

:   Finds min and max values of a float array.

---


### findMinMaxIntInt

```
public static int[] findMinMaxIntInt(int[][] f)
```

:   Finds min and max values of a int array.

---


### findMinMaxFloatArrays

```
public static float[] findMinMaxFloatArrays(float[][] f)
```

:   Finds min and max values of a float array.

---


### findClosestStartIndex

```
public static int findClosestStartIndex(int[] positions,
                                        int key)
```

:   Finds the index of the key in the int[] but wont preceed the int[index]

---


### findClosestEndIndex

```
public static int findClosestEndIndex(int[] positions,
                                      int key)
```

:   Finds the index of the key in the int[] but wont exceed the int[index]

---


### findClosestIndexToValue

```
public static int findClosestIndexToValue(int[] sortedValues,
                                          int key)
```

:   Returns the index of the closest values[index] to the key. Rounds up
    if the value is equi distant between two indexes. Will not return an
    index <0 or > length-1 .
    Don't forget to SORT!.
    If identical values are present, returns the smallest index containing the key.

---


### findClosestIndexToValue

```
public static int findClosestIndexToValue(float[] sortedValues,
                                          float key)
```

:   Returns the index of the closest values[index] to the key. Rounds up
    if the value is equi distant between two indexes. Will not return an
    index <0 or > length-1 .
    Don't forget to SORT!.
    If identical values are present, returns the smallest index containing the key.

---


### findStartStopIndexes

```
public static int[] findStartStopIndexes(int[] positions,
                                         int startValue,
                                         int stopValue,
                                         boolean notExact)
```

:   Finds the start and stop indexes given a sorted int[] of values and two values, one after the other,
    close together, otherwise just use Arrays.binarySearch. Good for huge int[] arrays. Set the notExact
    boolean to true if the values are potentially not exact.

---


### standardDeviationOfDifference

```
public static double standardDeviationOfDifference(int[] t,
                                                   int[] c)
```

:   Calculates the standard deviation of the difference between two int[]s
    of the same length.

---


### averageIntIntArray

```
public static double[] averageIntIntArray(int[][] ints)
```

:   Average int[][] values to double[], averages repeats [numRepeats][values]

---


### averageFloatArrays

```
public static double[] averageFloatArrays(float[][] ints)
```

:   Average float[][] values to double[], averages repeats [values][numRepeats]

---


### averageFloatArraysFlipped

```
public static double[] averageFloatArraysFlipped(float[][] ints)
```

:   Average float[][] values to double[], averages repeats [numChips][OligoValues].
    Assumes equal number of oligos.

---


### averageFloatArraysFlippedToFloat

```
public static float[] averageFloatArraysFlippedToFloat(float[][] ints)
```

:   Average float[][] values to double[], averages repeats [numChips][OligoValues].
    Assumes equal number of oligos.

---


### geoMeanFloatArraysFlipped

```
public static double[] geoMeanFloatArraysFlipped(float[][] ints)
```

:   Takes the geometric mean of float[][] values to double[], [numChips][OligoValues], returns the values
    in log base 2.
    Assumes equal number of oligos.

---


### averageFloatArraysFlippedToFloats

```
public static float[] averageFloatArraysFlippedToFloats(float[][] ints)
```

:   Average float[][] values to float[], averages repeats [numChips][OligoValues].
    Assumes equal number of oligos.

---


### geometricMeanSkipZeros

```
public static double[] geometricMeanSkipZeros(float[][] ints)
```

:   Takes a geometric average of float[][] values to double[], averages repeats [numChips][OligoValues].
    Assumes equal number of oligos. Skips zero values. Return in log base 10.

---


### geometricMean

```
public static double[] geometricMean(float[][] ints)
```

:   Takes a geometric average of float[][] values [numChips][OligoValues].
    Assumes no intensities <= 0.
    Returns log base 10 values.

---


### averageIntIntArray2

```
public static double[] averageIntIntArray2(int[][] ints)
```

:   Average int[][] values to double[], averages values [numRepeats][values]

---


### smoothScores

```
public static double[] smoothScores(double[] scores,
                                    int[] positions,
                                    int windowSize)
```

:   Uses a sliding window to smooth the scores applying a trimmed mean (drop 1 from ends) to the scores found within the window.
    The collection of values within the window is only an attempt to look windowSize/2 upstream and
    windowSize/2 downstream, not a requirement. Thus at either end of the scores possibly only 1/2 of
    the scores are used in making the calculation that is associated with the original score.

---


### meanIgnoreNaNInfinity

```
public static double meanIgnoreNaNInfinity(float[] f)
```

:   Calculates a mean on the non NaN and Infinity floats, return zero if no non zero values were found.

---


### windowAverageScores

```
public static double[] windowAverageScores(double[] scores,
                                           int windowSize)
```

:   Slides a window along an array, one index at a time, averaging the contents.

---


### windowAverageScoresIgnoreZeros

```
public static double[] windowAverageScoresIgnoreZeros(double[] scores,
                                                      int windowSize)
```

:   Slides a window along an array, one index at a time, averaging the contents, ignores zero values.

---


### windowAverageScoresIgnoreScore

```
public static float[] windowAverageScoresIgnoreScore(float[][] scores,
                                                     float scoreToIgnore,
                                                     int windowSize)
```

:   Slides a window along an array, one index at a time, averaging the contents, ignores zero values.

---


### windowAverageScoresIgnoreScore

```
public static float[] windowAverageScoresIgnoreScore(float[] scores,
                                                     float scoreToIgnore,
                                                     int windowSize)
```

:   Slides a window along an array, one index at a time, averaging the contents.

---


### windowAverageScores

```
public static float[] windowAverageScores(float[] scores,
                                          int windowSize)
```

:   Slides a window along an array, one index at a time, averaging the contents.

---


### trimmedMean

```
public static double trimmedMean(double[] sortedFloat,
                                 double fraction)
```

:   Trims the fraction off the top and bottom, returns the mean of the remainder, rounds trimming # up.
    If trim number resolves to 0 will proceed returning mean.

---


### trimmedMean

```
public static double trimmedMean(float[] sortedFloat,
                                 double fraction)
```

:   Trims the fraction off the top and bottom, returns the mean of the remainder, rounds trimming # up.
    If trim number resolves to 0 will proceed returning mean.

---


### trimmedMean

```
public static double trimmedMean(double[] sortedDouble,
                                 int trimNumber)
```

:   Makes a trimmed mean, note the trim number is not a % but the number of values to drop
    from the beginning and end of the ordered set. Set appropriately.
    Be sure to submit an ordered array!

---


### trimmedMean

```
public static double trimmedMean(float[] sortedFloat,
                                 int trimNumber)
```

:   Makes a trimmed mean, note the trim number is not a % but the number of values to drop
    from the beginning and end of the ordered set. Set appropriately.
    Be sure to submit an ordered array!

---


### arrayListOfDoubleToArray

```
public static double[] arrayListOfDoubleToArray(java.util.ArrayList dbl)
```

:   ArrayList of Double to double[]

---


### arrayListOfFloatToArray

```
public static float[] arrayListOfFloatToArray(java.util.ArrayList flt)
```

:   ArrayList of Float to float[]

---


### aveFoldDiffCombine

```
public static double aveFoldDiffCombine(int[] t,
                                        int[] c)
```

:   Calculates the average fold difference between two int[]s
    of the same length. Takes the mean of both arrays returns
    the ratio.

---


### aveFoldDiffIndividual

```
public static double aveFoldDiffIndividual(int[] t,
                                           int[] c)
```

:   Calculates the average fold difference between two int[]s
    of the same length. Calculates the ratio of each individually,
    then returns their average.

---


### ratio

```
public static double[] ratio(double[] t,
                             double[] c)
```

:   Returns the ratio of each pair.

---


### ratio

```
public static float[] ratio(float[][] t,
                            float[][] c)
```

:   Returns the ratio of each pair, averages replicas.

    :   **Parameters:**: `float[replicas][intensities]` -

---


### difference

```
public static float[] difference(float[][] t,
                                 float[][] c)
```

:   Returns the difference of each pair of average, aveT - aveC.

    :   **Parameters:**: `float[replicas][intensities]` -

---


### relativeDifferences

```
public static float[] relativeDifferences(float[][] t,
                                          float[][] c)
```

:   Returns the relative difference of each pair, averages replicas.

    :   **Parameters:**: `float[replicas][intensities]` -

---


### geometricMeanRatio

```
public static float[] geometricMeanRatio(float[][] t,
                                         float[][] c)
```

:   Returns the log2 ratio of each pair, takes a geometric mean of replicas.

    :   **Parameters:**: `float[replicas][intensities]` -

---


### logRatios

```
public static float[] logRatios(float[][] t,
                                float[][] c)
```

:   Returns the log2 ratio of each pair, averaging replicas.

    :   **Parameters:**: `float[replicas][intensities]` -

---


### ratio

```
public static double[] ratio(float[] t,
                             float[] c)
```

:   Returns the ratio of each pair.

---


### ratio

```
public static double[] ratio(int[] t,
                             double[] c)
```

:   Returns the ratio of each pair.

---


### ratio

```
public static double[] ratio(int[] t,
                             int[] c)
```

:   Returns the ratio of each pair.

---


### difference

```
public static double[] difference(int[] t,
                                  int[] c)
```

:   Returns the difference of each pair.

---


### difference

```
public static double[] difference(double[] t,
                                  double[] c)
```

:   Returns the difference of each pair.

---


### pooledSubArray

```
public static float[][] pooledSubArray(float[][] treatment,
                                       float[][] control,
                                       int startIndex,
                                       int stopIndex)
```

:   Builds a pooled from part of a float[replica][intensities] for treat and control.

    :   **Parameters:**: `treatment` - and control float[replicas][intensities]: `start` - and stop indexes are inclusive **Returns:**: float[treatment(0) or control(1)][pooled intensities]

---


### sam

```
public static float[] sam(float[] t,
                          float[] c,
                          double fudge)
```

:   Calculates the SAM d statistic, similar to a t-statistic with a bit of fudge added into the denominator to
    control for zero variance conditions. Set fudge to zero for standard unpaired T-Test.

    :   **Returns:**: float[3] samScore, diff of means, variance (w/o fudge).

---


### standardDeviation

```
public static double standardDeviation(int[] x)
```

:   Calculates standard deviation of an int[]

---


### standardDeviation

```
public static double standardDeviation(int[] x,
                                       double mean)
```

:   Calculates standard deviation of an int[] and mean.

---


### standardDeviation

```
public static double standardDeviation(float[] x)
```

:   Calculates standard deviation of an float[]

---


### standardDeviation

```
public static double standardDeviation(float[] x,
                                       double mean)
```

:   Calculates standard deviation of an float[] given a mean.

---


### standardDeviation

```
public static double standardDeviation(double[] x)
```

:   Calculates standard deviation of an double[]

---


### standardDeviation

```
public static double standardDeviation(double[] x,
                                       double mean)
```

:   Calculates standard deviation of a double[] and mean.

---


### standardError

```
public static double standardError(double[] x,
                                   double mean)
```

:   Calculates standard error of a double[] given it's mean.
    Spead savings

---


### medianAbsoluteDifference

```
public static double medianAbsoluteDifference(float[] x,
                                              float[] y)
```

:   Calculates the median absolute difference.

---


### calcMaxMedianAbsoluteDifference

```
public static double calcMaxMedianAbsoluteDifference(float[][] oligosValues)
```

:   Calculates the maximum median absolute difference between arrays of float where int[oligo index number][oligo intensity measurements]

---


### calcMeanMedianAbsoluteDifference

```
public static double calcMeanMedianAbsoluteDifference(float[][] oligosValues)
```

:   Calculates the mean of the median absolute differences between arrays of float where float[oligo index number][oligo intensity measurements]

---


### correlationCoefficient

```
public static double correlationCoefficient(int[] x,
                                            int[] y)
```

:   Calculates Pearson correlation coefficient, r, from two int[]s.
    Cannot have one int[] be uniform values, returns -2 if error.

---


### removeZeroValues

```
public static float[][] removeZeroValues(float[][] f)
```

:   Provide a float[2][0], will remove zeros from both and there matched
    pair.
    Does a pairwise zero removal, if either of the float arrays are zero
    both values are removed.

---


### formatPercentOneFraction

```
public static java.lang.String formatPercentOneFraction(double num)
```

:   Converts 0.345543 to 34.6%

---


### formatNumberOneFraction

```
public static java.lang.String formatNumberOneFraction(double num)
```

:   Converts a double ddd.dddddddd to sss.s

---


### formatNumber

```
public static java.lang.String formatNumber(double num,
                                            int numberOfDecimalPlaces)
```

:   Converts a double ddd.dddddddd to a user determined number of decimal places right of the .

---


### doubleArrayToString

```
public static java.lang.String doubleArrayToString(double[] d,
                                                   int numberOfDecimalPlaces,
                                                   java.lang.String delimiter)
```

:   Converts an array of double to a String with a defined number of decimal places and a delimiter.

---


### doubleArrayToStringOnlyMax

```
public static java.lang.String doubleArrayToStringOnlyMax(double[] d,
                                                          int maxNumDec,
                                                          java.lang.String delimiter)
```

:   Converts an array of double to a String with a maximal number of defined decimal places and a delimiter. Min isn't set.

---


### floatArrayToString

```
public static java.lang.String floatArrayToString(float[] f,
                                                  java.lang.String delimiter)
```

:   Converts to String using delimiter.

---


### intArrayToString

```
public static java.lang.String intArrayToString(int[] d,
                                                java.lang.String delimiter)
```

:   Converts an array of int to a String seperated by the delimiter.

---


### stringArrayToInts

```
public static int[] stringArrayToInts(java.lang.String s,
                                      java.lang.String delimiter)
```

:   Given a String of ints delimited by something, will parse or return null.

---


### stringArrayToFloat

```
public static float[] stringArrayToFloat(java.lang.String s,
                                         java.lang.String delimiter)
```

:   Given a String of floats delimited by something, will parse or return null.

---


### median

```
public static double median(double[] sorted)
```

:   Calculates Median of a sorted double[]. Copied code from WWW.

---


### median

```
public static double median(int[] sorted)
```

:   Calculates Median of a sorted int[].

---


### median

```
public static double median(short[] sorted)
```

:   Calculates Median of a sorted short[]. Copied code from WWW.

---


### median

```
public static double median(float[] sorted)
```

:   Calculates Median of a sorted float[]. Copied code from WWW.

---


### mean

```
public static float mean(float[] t)
```

:   Averages a float array.

---


### mean

```
public static float[] mean(float[] one,
                           float[] two)
```

:   Averages two float[]s .

---


### mean

```
public static float[][] mean(float[][] one,
                             float[][] two)
```

:   Averages two float[][]s, assumes a square.

---


### mean

```
public static double mean(int[] t)
```

:   Averages a int array.

---


### mean

```
public static double mean(double[] t)
```

:   Averages a double array.

---


### meanIntegers

```
public static int meanIntegers(java.util.ArrayList Integers)
```

:   Averages an ArrayList of Integer objects.

---


### meanDoubles

```
public static java.lang.String meanDoubles(java.util.ArrayList Doubles)
```

:   Averages an ArrayList of Double objects.

---


### findHighestInt

```
public static int findHighestInt(int[] ints)
```

:   Finds and returns the biggest int in an int[].

---


### findHighestFloat

```
public static float findHighestFloat(float[] ints)
```

:   Finds and returns the biggest float in an float[].

---


### sumIntArray

```
public static int sumIntArray(int[] ints)
```

:   Sums an int array

---


### sumArray

```
public static float sumArray(float[] array)
```

:   Sums a float array

---


### modeOfHistogram

```
public static int[] modeOfHistogram(int[] ints)
```

:   Finds mode of a int[] histogram array, assumes one peak, returns the index position, value/frequency.


---


|  |  |  |
| --- | --- | --- |
| **Overview** \| **Package** \| **Class** \| **Use** \| **Tree** \| **Deprecated** \| **Index** \| **Help** | |  |
| **PREV CLASS**   **NEXT CLASS** | **FRAMES**    **NO FRAMES**     **All Classes** |  |
| SUMMARY: NESTED \| FIELD \| CONSTR \| METHOD | DETAIL: FIELD \| CONSTR \| METHOD |  |


---
